# Molecular Mechanisms and Translational Therapies for Human Epidermal Receptor 2 Positive Breast Cancer

**DOI:** 10.3390/ijms17122095

**Published:** 2016-12-14

**Authors:** Quanxia Lv, Ziyuan Meng, Yuanyuan Yu, Feng Jiang, Daogang Guan, Chao Liang, Junwei Zhou, Aiping Lu, Ge Zhang

**Affiliations:** 1 Institute for Advancing Translational Medicine in Bone & Joint Diseases, School of Chinese Medicine, Hong Kong Baptist University (HKBU), Hong Kong 999077, China; 15484580@life.hkbu.edu.hk (Q.L.); 15484572@life.hkbu.edu.hk (Z.M.); yu.yy01@hotmail.com (Y.Y.); jiangfenghz@163.com (F.J.); guandg2012@gmail.com (D.G.); liangchao512@163.com (C.L.); waynebo@163.com (J.Z.); 2 Institute of Precision Medicine and Innovative Drug Discovery, HKBU (Haimen) Institute of Science and Technology (IST), Haimen 226133, China; 3 The State Key Laboratory Base of Novel Functional Materials and Preparation Science, Faculty of Materials Science and Chemical Engineering, Ningbo University, Ningbo 315211, China

**Keywords:** molecular mechanism, translational therapy, HER2 positive breast cancer, diagnostic tests, monoclonal antibodies, small molecular inhibitors, antibody–drug conjugates

## Abstract

Breast cancer is the second leading cause of cancer death among women. Human epidermal receptor 2 (HER2) positive breast cancer (HER2+ BC) is the most aggressive subtype of breast cancer, with poor prognosis and a high rate of recurrence. About one third of breast cancer is HER2+ BC with significantly high expression level of HER2 protein compared to other subtypes. Therefore, HER2 is an important biomarker and an ideal target for developing therapeutic strategies for the treatment HER2+ BC. In this review, HER2 structure and physiological and pathological roles in HER2+ BC are discussed. Two diagnostic tests, immunohistochemistry (IHC) and fluorescent in situ hybridization (FISH), for evaluating HER2 expression levels are briefly introduced. The current mainstay targeted therapies for HER2+ BC include monoclonal antibodies, small molecule tyrosine kinase inhibitors, antibody–drug conjugates (ADC) and other emerging anti-HER2 agents. In clinical practice, combination therapies are commonly adopted in order to achieve synergistic drug response. This review will help to better understand the molecular mechanism of HER2+ BC and further facilitate the development of more effective therapeutic strategies against HER2+ BC.

## 1. Introduction

Breast cancer is one of the most common cancers and the second leading cause of cancer death among women of all races [1]. According to the US breast cancer statistics, 12% of women and 0.1% of men in the US will develop invasive breast cancer during their lifetimes. In 2016, approximately 246,660 and 2600 new cases of invasive breast cancer are expected to be diagnosed in American women and men, respectively. Apart from lung cancer, death rates of breast cancer among women in the US are higher than those of any other cancer. Luckily, with the development of early stage diagnostic technology and increased awareness, the death rates caused by breast cancer have been decreasing since 1989. However, nearly 40,450 women diagnosed with breast cancer died in 2015 [2]. Breast cancers can be divided into four subtypes: luminal A (Estrogen Receptor (ER)+, Progestogen Receptor (PR)+, HER2− and Ki67 (which is a proliferation marker) <14%), luminal B (ER+, PR+, HER2− and Ki67 ≥14% or ER+, PR+, HER2+), basal-like (ER−, PR− and HER2−), and HER2 positive breast cancer (HER2+, ER− and PR−). Due to these complex molecular subtypes, it can be challenging to accurately diagnose and efficiently cure all different types of breast cancers [3].

Human epidermal growth factor receptor 2 (HER2) positive breast cancer (HER2+ BC), which belongs to a subtype of breast cancer with *HER2* gene amplification and HER2 protein overexpression, accounts for about 25%–30% of all breast cancers [4,5]. With aggressive biological behavior and poor clinical outcome, HER2+ BC is often associated with significantly shorter disease-free survival and worse overall survival rates than other subtypes of breast cancer. HER2 is a transmembrane protein with a molecular weight of 185 kDa. It plays a vital role in the regulation of cell growth, survival and differentiation [6]. The overexpression of HER2 favors cell proliferation by inhibiting cell apoptosis, which therefore leads to malignant tumors [7].

Accurately subtyping of the breast cancers is necessary to better identify molecular-based therapies. The expression level of HER2 is the critical indicator for breast cancer classification. Immunohistochemistry (IHC) and fluorescent in situ hybridization (FISH) are two commonly used methods in the clinic for evaluating the expression level of HER2. IHC is often utilized as the screening test to detect the expression levels of HER2 protein. In some ambiguous cases, the IHC results should be further validated and confirmed by FISH, which is more sensitive and reliable [8].

There are four mainstay HER2 targeted therapeutic methods for the treatment of HER2+ BC, including monoclonal antibodies, small molecule tyrosine kinase inhibitors, antibody–drug conjugates (ADC) and other emerging anti-HER2 agents. Trastuzumab (Herceptin^®^, Genetech) and pertuzumab (Perjeta^®^, Genetech) are the two different Food and Drug Administration (FDA) approved monoclonal antibody drugs against the extracellular domain of HER2. Trastuzumab is the first line and the most preferred antitumor drug for HER2+ BC. Though many studies have proved the satisfactory therapeutic efficacy of trastuzumab [9,10], some HER2+ BC patients showed intrinsic or acquired resistance to it [11]. Hence, novel anti-HER2 agents are continuing to be developed. Lapatinib (Tykerb^®^, GlaxoSmithKline) is a small molecule tyrosine kinase inhibitor, which is the second FDA approved HER2 targeted drug after trastuzumab. Afatinib (BIBW-2992, Boehringer Ingelheim) and neratinib (HKI-272, Puma Biotechnology) are another two dual tyrosine kinase inhibitors for HER2+ BC treatment. Trastuzumab–emtansine (T-DM1, Genetech) is an antibody drug conjugate targeting HER2 combining an anti-microtubule cytotoxic chemical agent with monoclonal antibody trastuzumab. In clinical practice, in order to achieve synergistic drug response and higher therapeutic efficacy, combination therapies are mostly adopted, for example the combination of trastuzumab with pertuzumab, trastuzumab with lapatinib, and combination of anti-HER2 agents with chemotherapeutic agents [6,12,13,14].

In this review, the biological function of HER2 and its molecular mechanism for tumorigenesis, HER2 specific diagnostic and the current therapeutic strategies for HER2+ BC are discussed. This review will help to better understand the molecular mechanism of HER2+ BC and further facilitate the development of more effective therapeutic strategies against HER2+ BC.

## 2. HER2 Biology and Its Role in Breast Cancer

### 2.1. Structure of HER2 and Its Physiological Role in Signaling Pathways

#### 2.1.1. Structure of HER2

**Figure 1:**
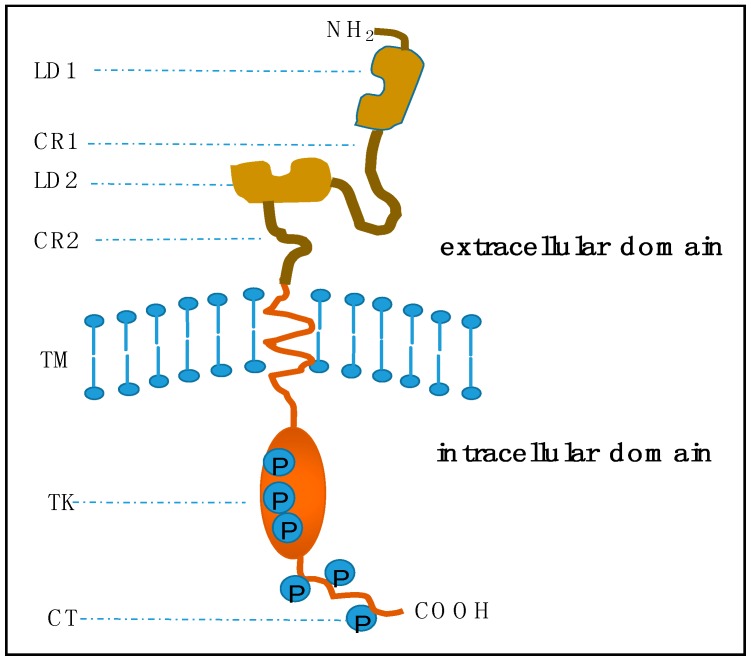
Basic structure of epidermal growth factor receptor (EGFR) transmembrane proteins. In the extracellular domain, LD1 and LD2 are two repeated ligand binding domains. CR1 and CR2 are two repeated cysteine rich regions. TM indicates the short transmembrane spanning sequences. In the intracellular domain, TK is a catalytic tyrosine kinase, and CT is the carboxyl-terminal tail. Circled Ps are the phosphorylation sites within the TK and CT regions. This figure is revised based on the review of the oncogene human epidermal growth factor 2 (*HER2*) contributed by Moasser [17].

Human epidermal growth factor receptor 2 (*HER2*/*neu*, ErbB2) is a 185 kDa transmembrane glycoprotein encoded by the *HER2*/*neu* oncogene located at chromosome 17q. It belongs to the epidermal grow factor receptor (EGFR) family of epithelial tyrosine kinases, which also includes other three distinct receptors: EGFR (ErbB1), HER3 (ErbB3), and HER4 (ErbB4). Proteins in the EGFR family are all transmembrane proteins sharing a common basic molecular structure: an extracellular ligand-binding domain with an amino-terminal, a single transmembrane spanning region and an intracellular cytoplasmic domain with tyrosine kinase activity (Figure 1) [15,16]. The extracellular domain consists of four parts: two repeated ligand binding domains (LD1 and LD2) responsible for ligand recognition, and cysteine rich sequences (CR1 and CR2) providing a framework to orientate LD regions. The intracellular domain can be divided into two regions: a catalytic tyrosine kinase (TK) domain with phosphorylation sites and a carboxyl-terminal tail (CT) [17].

#### 2.1.2. Role in Signaling Pathways

Binding of receptor specific ligands to the extracellular domain of EGFR receptors could induce homo- and heterodimerization of these receptors. Different from other EGFR family members, HER2 does not have identified ligands and it constitutively exists in an activated conformation. It is considered as the most preferred dimerization partner with the most potent kinase catalytic activity. Therefore, HER2 engaged dimerization is more effective on the regulation of cellular processes [18,19]. The heterodimer formed by HER2 and HER3 is the most active signaling complex among other pairs of EGFR receptors [17,20,21].

**Figure 2:**
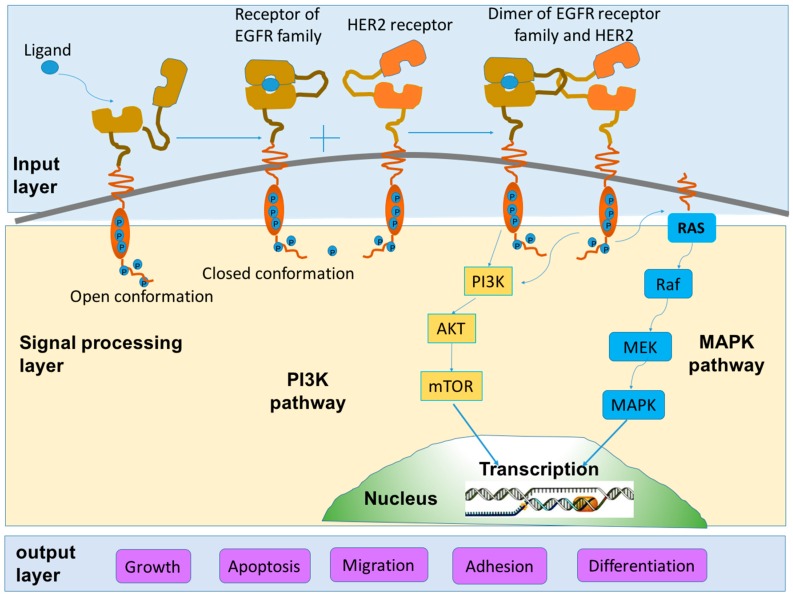
Schematic diagram of HER2 signaling pathways. Upon ligand binding, dimerization between receptors of EGFR family and HER2 receptor is induced. The homodimers or heterodimers thereafter stimulate a serial of signaling cascades. Among various signaling pathways, the phosphatidyl inositol 3-kinase (PI3K) and mitogen-activated protein kinase (MAPK) pathways are the two major and most studied pathways which take a pivotal role in tumor proliferation and anti-apoptosis. The whole signal transduction process can be divided into three sections: signal input (ligand-binding and dimerization), signal processing (a series of signaling cascades) and signal output (corresponding cellular processes). The scheme is modified based on two works contributed by Yarden et al. [15] and Tai et al. [16], respectively.

The formation of heterodimers or homodimers thereafter activates the intracellular tyrosine kinase and triggers the autophosphorylation of specific tyrosine residues. The phosphorylation of tyrosine residues in turn recruits adaptor proteins or enzymes to initiate a succession of signaling cascades to regulate cellular processes [22]. These signaling cascades can be transduced via at least two distinct pathways: phosphatidyl inositol 3-kinase (PI3K)-Akt and Ras/Raf/mitogen-activated protein kinase (MAPK). Different intracellular signaling cascades are induced by different types of dimers [23]. The induction of PI3K signaling activities is stimulated by the heterodimer composed of HER2 and HER3. However, as for Ras/Raf/MAPK signaling pathway, it can be activated by all of the dimers containing HER2 (HER1/HER2, HER2/HER2, HER2/HER3 and HER2/HER4) [16]. The PI3K and MAPK signaling pathways are the two most studied key signaling transduction pathways that favor cell proliferation by inhibiting cell apoptosis. Figure 2 shows the scheme of the two HER2 signaling pathways.

In normal cells, these signaling cascades will be terminated primarily relying on endocytosis of the complexes formed by EGFR receptors and their corresponding ligands. Then the EGFR receptors may have two destinations: recycling to the cell surface or degradation by various enzymes. Based on these processes, a dynamic balance of the physiologic outcomes, which includes cell division, survival, proliferation and apoptosis, will be maintained. Under normal circumstances, these signaling processes are essential for normal cell growth and will not lead to tumor growth [22].

### 2.2. Tumorigenic Action of HER2

In normal cells, HER2 plays a vital role in various cellular processes and the expression level of HER2 remains stable. However, when overexpression of HER2 occurs for various reasons, it will lead to tumorigenesis and metastasis [7].

As mentioned above, HER2 engaged dimerization is more effective on the regulation of cellular processes especially in promoting cell proliferation by inhibiting cell apoptosis. Several molecular rationales may illustrate the phenomenon. First, HER2 is the priority choice for other receptors in EGFR family members to form heterodimers [19]. Overexpression of HER2 makes excessive HER2 receptors available for ligand-activated HER1/HER3/HER4 binding to form extra heterocomplexes [24]. Second, HER2 may strengthen the affinity of ligand-binding for other receptors to their corresponding ligands, probably by slowing down the dissociation rate of ligands from the formed active heterodimers. Furthermore, HER2 may weaken the specificity of its heterodimerization partners, which enables HER2 to pair with a broader spectrum of EGFR analogues [25]. Consequently, HER2-containing heterodimers are capable of responding for a prolonged time with a strong signal. Third, HER2 engaged dimerization can activate both of the key signaling pathways: the cell proliferative RAS/Raf/MAPK pathway and the cell survival PI3K/Akt pathway. Finally, HER2-containing heterodimers may escape from the inactivation processes by decreasing the rate of internalization or degradation of HER2 dimers, and recycling to cell surface rather than going into a degradative pathway [22]. All these processes caused by the overexpression of HER2 may disrupt the dynamic balance of various cellular processes and lead to uncontrollable tumor growth.

Taken together, the role of HER2 in the regulation of cellular processes can be summarized in a simple way. When the *HER2*/*neu* gene expresses HER2 protein normally, only appropriate numbers of HER2 heterodimers will be established, and therefore the signaling responses to these growth factors will be at normal levels. However, when amplification of HER2/neu gene occurs, it induces the overexpression of HER2 protein (usually 10–100-fold of greater than the nearby normal cells). Then an excessive amount of HER2-containing heterodimers will be formed which enhances the signaling responses to growth factors [26]. Finally, malignant growth and tumorigenesis appears [23].

#### 2.2.1. HER2 and Breast Cancer

Among all the cancers related with *HER2* amplification and HER2 overexpression, breast cancer is the most widely studied type. HER2 positive breast cancer (HER2+ BC), with HER2 gene amplification and HER2 protein overexpression, accounts for about 25%–30% of all breast cancers [4,5]. With aggressive biological behavior, chemotherapy resistance and poor prognosis, HER2+ BC is considered to be one of the toughest subtypes of all breast cancers [27,28,29]. It is reported that the overall survival rate as well as relapse time for HER2+ BC patients are remarkably shorter than those with other subtypes of breast cancers [16].

There are several cellular mechanisms that may underlie the poor prognosis in patients with HER2+ BC [22,30]. Firstly, overexpression of HER2 in HER2+ BC may strengthen the metastatic properties of tumor cells, including invasion, angiogenesis, stronger survival and greater proliferation [31]. Secondly, HER2+ BC has greater chance to be resistant to some therapies, such as chemotherapy and hormone therapy. The resistance may therefore lead to poor and low drug response to these therapies [32]. Moreover, a high percentage of S-phase cells in HER2+ BC samples indicates that overexpression of HER2 is in favor of cell proliferation. Additionally, overexpression of HER2 in HER2+ BC may be related to the larger tumor size and aneuploidy [22].

#### 2.2.2. HER2 and Other Cancers

Apart from HER2+ BC, overexpression of HER2 is also frequently found in gastric cancer, ovarian cancer and prostate cancer. Gastric cancer is the second leading cause of cancer death worldwide. HER2 overexpression in gastric cancer was first described in 1986 [33]. Thereafter, accumulated evidences revealed the correlation of HER2 overexpression and poor clinical outcomes [34,35,36,37]. HER2-overexpressing gastric cancer approximately varies from 6% to 35% [38]. Ovarian cancer is the most common cause of gynecological cancer death. The percentage of HER2 overexpression in ovarian cancer is estimated in the range of 9%–32% [39,40,41]. HER2 overexpression is also found in prostate cancer and many efforts have been made to study the HER2 expression in prostate cancer [42,43].

## 3. Diagnosis of HER2+ BC

The 2007 American Society of Clinical Oncology (ASCO) guidelines recognized that HER2 is an important prognostic, predictive, and therapeutic biomarker in invasive breast cancer. Evaluation of HER2 status in breast cancer has prognostic and therapeutic response value. To be more specific, HER2 status may facilitate the choice of appropriate molecular therapy by predicting the response to chemotherapy, hormonal therapy and anti-HER2 therapy [44]. Therefore, it is necessary to determine HER2 status in every primary breast cancer either at the time of diagnosis or recurrence to guide therapy. Currently, there are various methods for determining HER2 status, such as IHC, enzyme-linked immunosorbent assay (ELISA) analysis and Western blot test for HER2 protein overexpression, FISH, chromogenic in situ hybridization (CISH), silver in situ hybridization (SISH), Southern blot and polymerase chain reaction (PCR) for *HER2*/*neu* gene amplification [44,45,46,47]. Of all these tests, IHC and FISH are the two most frequently used methods to assess HER2 status. They have the common property of being able to correlate HER2 expression to morphologic features [48]. About 80% of HER2 evaluation begins with IHC, which is used primarily as a screening test [8].

**Figure 3:**
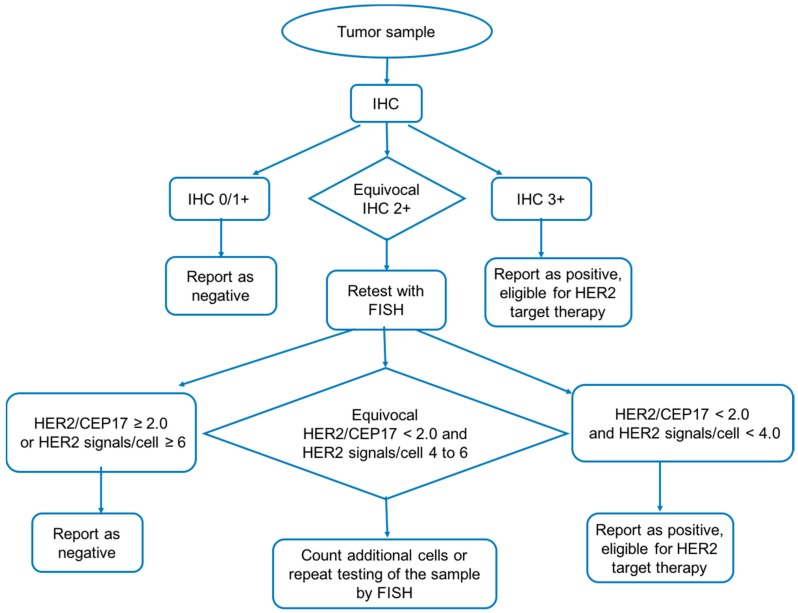
Workflow of HER2 positive breast cancer diagnosis. Tumor samples are initially tested by immunohistochemistry (IHC). Then the samples are divided into three subtypes based on the slide scores of IHC: negative report cases (IHC 0/1+), equivocal cases (IHC 2+) and positive cases (IHC 3+). The equivocal samples will be retested by FISH to verify its HER2 expression more accurately. The figure is a modified version based on Bilous’s work [44].

Most national testing guidelines suggest the following testing workflow to diagnosis of HER2+ BC (Figure 3). Tumor samples are initially tested by IHC which acts as screening test. Then the samples are divided into three subtypes based on the slide scores of IHC: negative report cases (IHC 0/1+), equivocal cases (IHC 2+) and positive cases (IHC 3+). The equivocal samples will be retested by FISH to verify their HER2 expression more accurately [44]. Positive cases indicate patients are eligible for anti-HER2 therapies [49,50].

### 3.1. Immunohistochemistry

There are many advantages of IHC testing to determine the HER2 status; it is fast, widely used, relatively cheap, easy to preserve species and convenient to carry out by using common microscope. Therefore, it becomes the most frequently used primary technique to assess HER2 status. The detection process is as follows. Firstly, the slides of tumor samples are incubated with a specific antibody directly against HER2 protein. Then, the free unbound antibodies are washed away and an enzyme-conjugated secondary antibody against the primary antibody is added. Finally, the complex is made visible with a chromogen. A colorimetric relationship will be established between the number of HER2 proteins on the cell surface and the distribution as well as intensity of the immune stain. The scoring system of the staining results is following the DAKO (Glostrup, Denmark) guidelines (Table 1) [51]. In the scoring system, tumor cells containing fewer than 20,000 HER2 receptors would show no staining (negative, IHC 0); cells containing about 100,000 HER2 receptors would show weak or incomplete membrane staining with less than 10% of the cells stained (IHC 1+); cells containing approximately 500,000 HER2 receptors would show light to moderate complete membrane staining in more than 10% of the cells (IHC 2+) and cells containing approximately 2,300,000 HER2 receptors would show strong and complete membrane staining in more than 30% of the tumor cells (IHC 3+) [47,49].

On the other hand, IHC testing has some limitations, for example the effect caused by the pre-analytic, analytic and post-analytic processes. There is no uniform control for time, type of tissue fixation, and temperature of paraffin embedding, and there is no standard for processing the tissue samples, all of which may result in fluctuation of the IHC results of the same specimens. Moreover, reduced immuno-reactivity for HER2 proteins to their corresponding antibodies may happen due to prolonged storage of unstained slides, causing the IHC scores to be negatively affected. Most importantly, IHC test is a semi-quantitative method, as it is based on a subjective determination of the intensity of the color reaction [52].

### 3.2. Fluorescent In Situ hybridization (FISH)

FISH is a more reliable, sensitive and accurate testing tool with less influence by pre-analytic and analytic variable factors. A major advantage of FISH compared to IHC is that the evaluation of HER2 status is much more quantitative and includes internal control cells which are located adjacent to tumor cells in the same tissue section for parallel comparison [50]. This method is used not only in the diagnosis of breast and gastric tumors, but also in monitoring the response to treatment for these tumors [53].

Three types of FISH assays are approved by the FDA, including PathVysion (Abbott Laboratories) and Dako PharmDx (Dako Corporation) FISH test which two use dual probes (one for HER2 and the other for the centromere 17) and the Ventana Inform (Ventana Medical Systems) which uses a single probe only for HER2. These testing methods share a similar testing procedure, which utilizes fluorescently labeled probes that are partly complementary to the *HER2*/*neu* gene [53]. After binding to complementary DNA of the tumor cells on the slide, the probes can be visible under a fluorescence microscope. Then the number of *HER2*/*neu* gene copies can be estimated. The ratio of *HER2*/*neu* gene to chromosome 17q centromere (CEP 17) will then be calculated [45]. According to current guidelines, the FISH results can be interpreted in a detailed and complex way (Table 2) [54].

However, FISH also has its limitations. It usually takes about two days and requires the use of expensive fluorescence microscopy. In addition, it requires extensive training and expertise to distinguish between normal cells and malignant cells from the FISH results [44]. Furthermore, it also takes much time to score the results and sometimes the scoring may be affected by the overlapping of nuclei. Additionally, the fluorescence may fade out overtime; the sample slides and kits should be carefully preserved and tested as soon as possible. Lastly, the prior protein digestion may affect the morphology of tumor samples, making it difficult to recognize. Therefore, FISH is not chosen as the primary screening test and it is mainly used in IHC 2+ group to confirm the HER2 status of the tumor samples [47,48].

## 4. Drugs Targeting HER2

**Figure 4:**
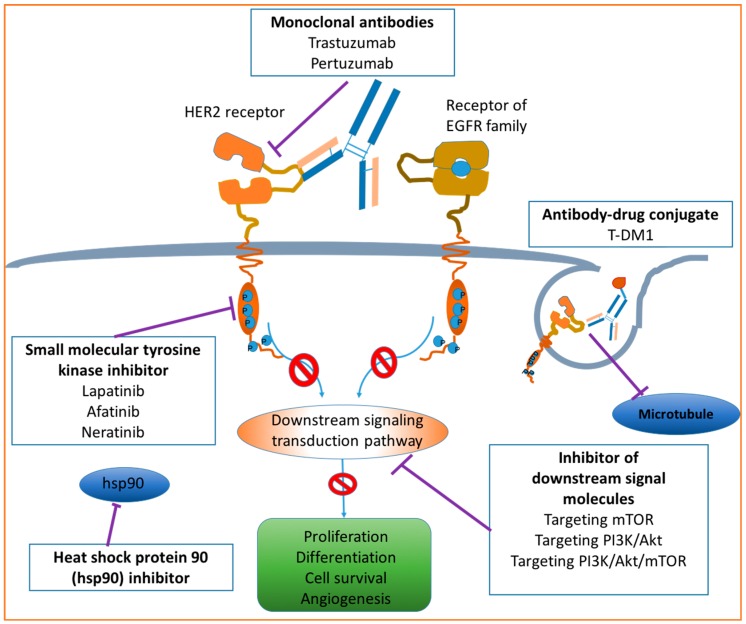
Molecular approaches for HER2+ BC therapy. Modified from the work contributed by Tsang [13]. Drugs targeting HER2 may include monoclonal antibodies, small molecular tyrosine kinase inhibitors, antibody–drug conjugates, heat shock protein 90 inhibitors and inhibitors of downstream signal molecules. T-DM1: Trastuzumab–emtansine.

Overexpression of HER2 is closely related to the development and progression of breast cancer. Therefore, HER2 becomes a critical target for developing therapeutic drugs against HER2+ BC. The development of anti-HER2 therapies has significantly improved the clinical outcome for patients with HER2+ BC [12]. In this mini-review, four major anti-HER2 drugs for HER2+ BC treatment are discussed, including monoclonal antibodies, small molecule tyrosine kinase inhibitors, ADC and other emerging anti-HER2 agents. Combination therapies using monoclonal antibodies with other drugs are also discussed. The acting pathways of involved molecular approaches for the treatment of HER2+ BC are briefly presented in Figure 4.

### 4.1. Monoclonal Antibodies

#### 4.1.1. Trastuzumab

Trastuzumab (Herceptin^®^, Genetech) is the first humanized monoclonal antibody approved for the treatment of HER2+ BC by the FDA in 1998. With the advent of trastuzumab, the prognosis of patients with HER2+ BC both in metastatic and adjuvant settings has been dramatically improved. Several clinical trials have shown that trastuzumab improves overall survival (OS) in metastatic breast cancer [9,55], increases pathological complete response (pCR) in the neoadjuvant setting [56], and improves disease-free survival (DFS) and OS in the adjuvant setting [57,58].

##### Mechanism of Action

**Figure 5:**
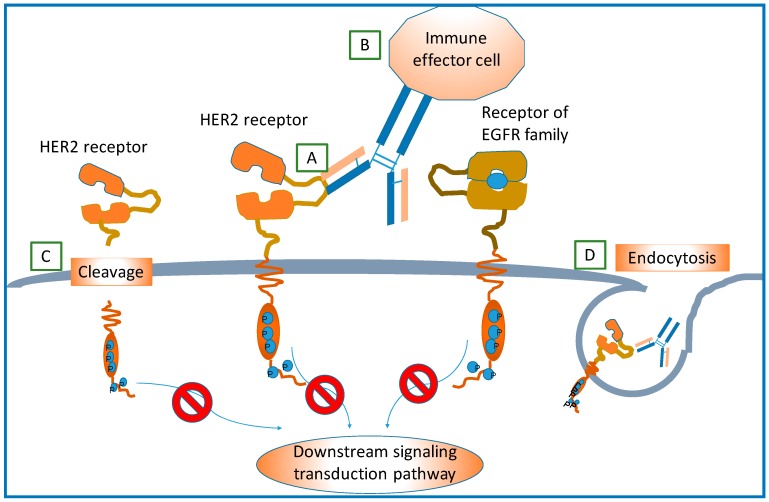
Potential action mechanisms of trastuzumab targeting HER2 receptor. (**A**) Blocking of the dimerization of HER2 and other EGF receptors; (**B**) Role of antibody-dependent immune-mediated response; (**C**) Binding of trastuzumab and HER2 may prevent HER2 extracellular domain from cleavage or shedding, which would further inhibit downstream signaling transductions and promote cell apoptosis; and (**D**) Endocytosis of HER2 receptor conjugated with trastuzumab. Reproduced based on Hudis’s work [59].

The mechanisms through which trastuzumab exerts its effects are not completely clear, but it is believed to be involved in the prevention of the formation of HER2-containing heterodimers, antibody-dependent cell-mediated cytotoxicity (ADCC), disruption of downstream signaling pathways, inhibition of cleavage of HER2, and promoting endocytosis of HER2 receptors [59,60]. These possible mechanisms of action of trastuzumab on HER2 are shown in Figure 5.

##### Mechanisms of Resistance

Though trastuzumab exerts its effect quite successfully in the treatment of early and advanced HER2+ BC, a proportion of patients have intrinsic or acquired resistance to it. In general, there are four proposed mechanisms for resistance to trastuzumab [61,62,63,64].

First, the interaction between receptor and antibody is blocked. Obstacles preventing trastuzumab binding to HER2 also explain the resistance to some extent. Several studies reported that MUC4, also a transmembrane protein, may mask the epitope on HER2. Hence, trastuzumab has difficulty to access and bind to HER2. Therefore, the efficacy of trastuzumab may be significantly reduced [65,66].

Second, the immune system fails to respond. As is mentioned above, ADCC may take a non-negligible role in the antitumor action of trastuzumab. If polymorphisms and other dysfunctions of Fc receptor make it fail to trigger an immune-mediated mechanism, this would reduce the ADCC response to trastuzumab, leading to resistance to trastuzumab [11].

Third, downstream signaling pathways are upregulated. Phosphatase and tensin homolog (PTEN) loss, presence of p95 (extracellular receptor cleavage forming truncated form of HER2 p95) [67,68] and presence of excess ligands may belong to this category of hypothetical resistance mechanisms. They all upregulate downstream signaling pathways that promote cell proliferation and inhibit apoptosis even though HER2 dimerization is blocked by trastuzumab. A study found that activation of PTEN helped trastuzumab to exert its antitumor activity. Deficiency of PTEN may be a significant predictor for trastuzumab resistance [69]. As an evidence, it is reported that a high level of p95 HER2 in primary tumor cells has significant correlation with reduced five-year disease-free survival (DFS) (*p* < 0.0001) [70]. Presence of excess ligands can also lead to resistance. It is reported that excess ligands may stimulate formation of more HER2-containing heterodimers and drive cells towards uncontrollable growth with reduced apoptosis. In this kind of tumor cell lines, trastuzumab may fail its mission or be less efficient [71].

Fourth, alternative signaling pathways are activated. Even though trastuzumab successfully recognizes and acts on HER2 preventing the signaling transduction pathways induced by HER2-containing dimers, alternative pathways unrelated with HER2 can be activated by other factors, such as insulin-like growth factor-I receptor (IGF-IR) [72].

#### 4.1.2. Pertuzumab

Pertuzumab (Perjeta^®^, Genetech) is the second humanized monoclonal antibody for anti-HER2 therapy approved by the FDA in 2013. Like trastuzumab, it exerts its function by binding with an epitope on the extracellular domain of HER2 receptors, therefore preventing the formation of HER2-containing heterodimers. However, pertuzumab could form a complex with HER2 on the heterodimerization interface, which has different epitopes from trastuzumab [73,74]. Based on the in vitro studies and clinical data, pertuzumab is superior in disrupting the formation of HER1 and HER2 heterodimer and HER2 and HER3 heterodimer [75]. However, pertuzumab as single agent for the treatment of HER2+ BC without any chemotherapy has limited efficacy [76]. Nevertheless, when pertuzumab is used in combination with trastuzumab providing a more complete blockade of the HER2 signaling pathway, it greatly optimizes the antitumor effects [76,77]. In this aspect, the combination has partially solved the drug resistance when trastuzumab failed to bind to HER2 due to one site mutation of HER2. However, it cannot address other situations of resistance, such as activation of alternative signaling pathways and upregulation of downstream signaling.

#### 4.1.3. Antibody Drugs for HER2+ BC in Ongoing Clinical Trials

Besides the two existing FDA-approved monoclonal antibodies for HER2+ BC therapy, there are some novel monoclonal antibodies directed against HER2. Margetuximab (MGAH22, MacroGenics) is one of them, showing great promise. It is an Fc-optimized chimeric monoclonal antibody with enhanced ADCC [78]. Currently margetuximab is under its phase III clinical trial enrolling patients with HER2+ BC (NCT02492711) [79]. Another studied antibody is a bispecific antibody fusion protein named MM-111 (Merrimack Pharmaceuticals, Cambridge, MA, USA). MM-111 is developed by linking modified human serum albumin with anti-HER2 and HER3 single chain antibody moieties together. It is postulated that MM-111 could anchor to HER2 and block heregulin-induced activation of HER3 to treat HER2/HER3 driven breast cancer. Thus, it may be a complement for the current anti-HER2 therapy for treatment of HER2+ breast cancer [80,81].

### 4.2. Small Molecule Tyrosine Kinase Inhibitors

Monoclonal antibodies exert their functions by binding to the extracellular domain of HER2 receptor, which prevents the dimerization of HER2 and other EGF receptors, while tyrosine kinase inhibitors (TKIs) bind to the intracellular tyrosine kinase domain of HER2 to prevent the phosphorylation of tyrosine kinase and thus inhibit the activation of downstream signaling pathways.

#### 4.2.1. Lapatinib

**Figure 6:**
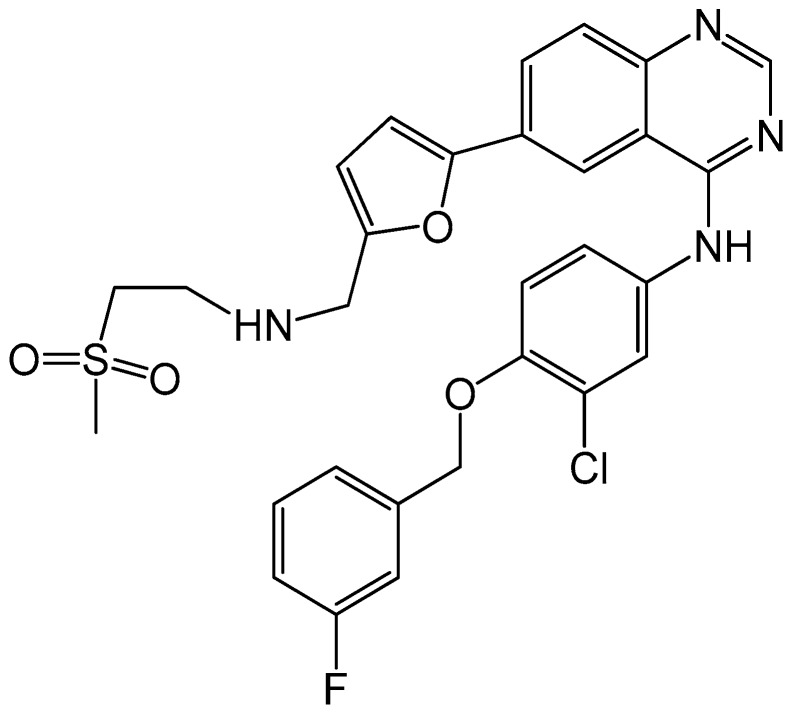
Chemical structure of lapatinib, with the chemical formula C_29_H_26_ClFN_4_O_4_S and a molecular weight of 581.0575 g/mol.

Lapatinib (Tykerb^®^, GlaxoSmithKline) is the second anti-HER2 targeting agent, approved by the FDA in 2005. It is an orally active, reversible and dual small molecular inhibitor of HER2 and HER1 with prolonged inhibition of tyrosine phosphorylation in tumor cells [82,83,84]. Figure 6 shows the chemical structure of lapatinib. A previous study found lapatinib showed high efficacy to halt tumor progression when used as a first-line therapy for HER2+ BC. The result showed about 24% patients (*n* = 138) who received treatment of lapatinib for 17.6 weeks demonstrated an overall response (OR) of 31%, and progression-free survival (PFS) rate of 63% at Month 4 and 43% at Month 6. The most common adverse events caused by lapatinib were diarrhea, rash, pruritus, and nausea. However, these events were primarily graded at mild level as 1 or 2 [85]. This study indicated that lapatinib is clinically active and well tolerated, which makes it reasonable to further evaluate the possibility of lapatinib to be used as first-line agent in treatment of HER2+ BC.

Several postulated acting mechanisms could be concluded based on studies [16]. First, it could target the tyrosine kinase domain of HER2 and HER1 to inhibit the downstream signaling transduction. Second, it could also interrupt the activity of molecules involved in the HER2 activated downstream signaling transduction [86]. Moreover, it could induce cell apoptosis by inhibiting IGF-IR. Since lapatinib acts through different manners with trastuzumab, it may retain significant activity in patients with HER2+ BC who are resistant to trastuzumab [87]. Therefore, lapatinib in combination with monoclonal antibodies may produce a synergistic effect in antibody resistant cancers. As an example, it is reported that a combination therapy of lapatinib with anti-HER2 antibody enhanced the apoptosis in HER2+ BC cells [88]. Another study evaluating trastuzumab plus lapatinib for the treatment of HER2+ BC reported that the observed response rate (ORR) was 50% in the first-line; 57% and 40% of patients in the two cohorts gained clinical benefit; and it showed a higher ORR (in the range of 24%–35%) than that of using trastuzumab or lapatinib alone [89].

#### 4.2.2. Afatinib

**Figure 7:**
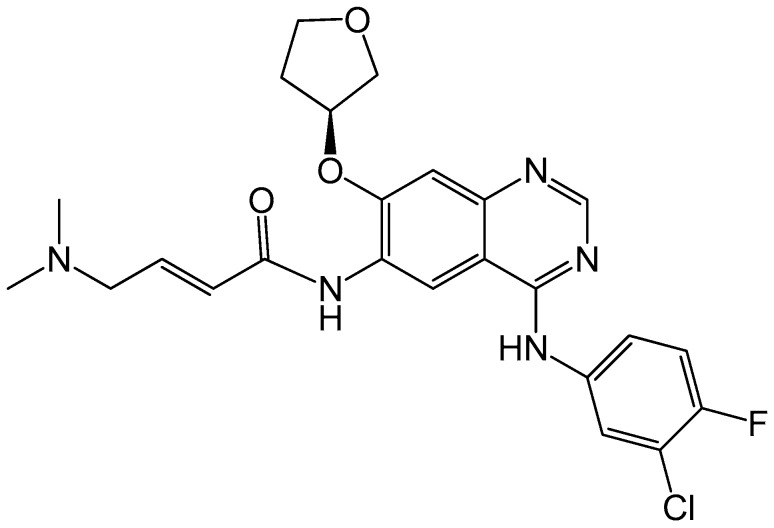
Chemical structure of afatinib, with the chemical formula C_24_H_25_ClFN_5_O_3_S and a molecular weight of 485.9384 g/mol.

Afatinib (BIBW-2992) (Boehringer Ingelheim) is an orally active irreversible dual inhibitor of EGFR and HER2. Its chemical structure is presented in Figure 7. In a phase II study, afatinib monotherapy was carried out in heavily pretreated HER2+ MBC patients in which partial response (PR) was 10% (*n* = 41) and stable disease was 37% (*n* = 37) [90]. Though afatinib has some antitumor activities in HER2+ BC, it is usually used in combination with other anti-HER2 agents, such as trastuzumab.

#### 4.2.3. Neratinib

**Figure 8:**
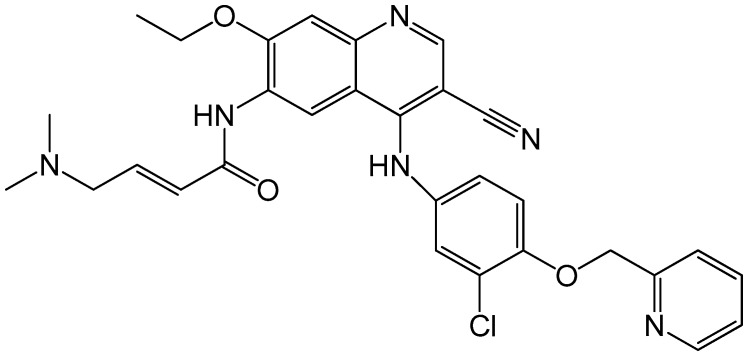
Chemical structure of neratinib, with the chemical formula C_30_H_29_ClFN_6_O_3_S and a molecular weight of 557.0427 g/mol.

Neratinib (HKI-272, Puma Biotechnology) is also an orally taken and active irreversible inhibitor of EGFR, HER2 and HER4 receptors. Figure 8 shows its chemical structure. It can inhibit the downstream signaling transduction induced by HER2 dimerization and thus prevent tumor cell proliferation. In a phase II open-label clinical trial, 240 mg of oral neratinib was administered to two cohorts of patients. One cohort was pretreated with trastuzumab (*n* = 66) and the other was not pretreated (*n* = 70). The overall response rate (ORR) was 24% and 56%, respectively. Efficacy results were better in the non-pretreated group, with PFS rate 78% in the trastuzumab native group and 59% in the trastuzumab pretreated group. Moreover, the study also reported that a majority of the recruited patients showed reduction in their tumor sizes [91]. Therefore, neratinib has great potential in showing substantial clinical activity.

### 4.3. Antibody–Drug Conjugates

#### 4.3.1. Ado–Trastuzumab Emtansine

**Figure 9:**
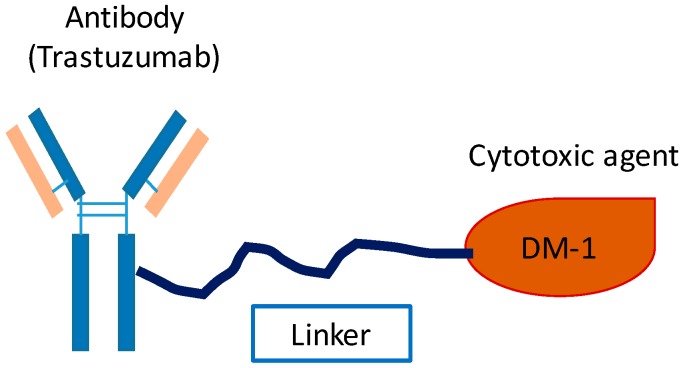
Molecular structure of T-DM1. The monoclonal antibody (trastuzumab) was conjugated with a cytotoxic agent (emtansine, which is also named DM1) through a thioether linker.

Ado–trastuzumab emtansine (T-DM1, Genentech) is an ADC which was approved by the FDA in 2013 [92]. The structure of T-DM1 is presented in Figure 9. Via a stable thioether linker, T-DM1 can be selectively delivered into tumor cells with HER2 overexpression. After a series of steps, the DM1 can be liberated in the cytoplasm, resulting in cell cycle arrest and apoptosis [93,94,95].

**Figure 10:**
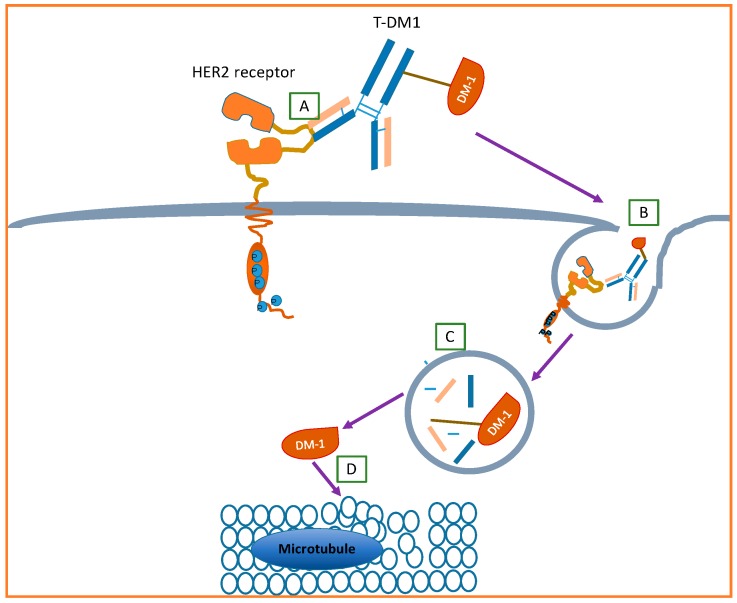
Potential action mechanism of T-DM1. (**A**) Trastuzumab specifically recognizes and binds to extracellular domain of HER2; (**B**) induced passive endocytosis of the formed complex of trastuzumab and HER2; (**C**) the complex may undergo a degradation process to separate the DM-1 from the complex; and (**D**) the liberated DM-1 exerts its cytotoxicity on the microtubule. Modified based on the work contributed by Martinez et al. [94].

The potential acting mechanism is shown in Figure 10. Initially, trastuzumab specifically recognizes and binds to the extracellular domain of HER2. Then, the interaction of the above step may induce passive endocytosis of the formed complex of trastuzumab and HER2. Next, the complex enters into the cytoplasm. Under the action of various enzymes in intracellular lysosomes, the complex may undergo a degradation process, which separates the DM-1 from the complex. Finally, the liberated DM-1 exerts its cytotoxicity on microtubules by inhibiting their formation and thus lead to cell apoptosis.

#### 4.3.2. A Novel Biparatopic ADC for HER2+ BC

Recently, a study constructed a novel HER2 directed ADC of a biparatopic antibody linked with a tubulysin-based microtubule inhibitor. It was found that this novel ADC showed a greater efficacy for HER2+ BC than T-DM1. The reported biparatopic antibody can recognize and bind to two distinct epitopes on the HER2 receptor. This interaction could induce clustering of HER2 which could then facilitate the internalization, lysosomal trafficking and degradation of the clustered HER2. Through thoughtful experimental design, the study also indicated that a broader group of patients (eligible or ineligible and relapsed/refractory for T-DM1 treatment) could benefit from this biparatopic ADC [96].

### 4.4. Other Emerging HER2 Targeted Agents

Heat shock protein 90 (HSP90) inhibitors are novel therapeutic approach for HER2+ BC. HSP90 is a molecular chaperone which is necessary for maintaining the stability and function of proteins. When HSP90 is inhibited by its corresponding inhibitors, its client protein will be unstable and eventually undergo degradation by proteinases. HER2 is one of the most sensitive client proteins of HSP90 [97]. High levels of HSP90 in breast cancer are reported to be associated with decreased survival rate [98]. HSP90 inhibitor can inhibit the activity of HSP90 and thus promote the degradation of HER2 receptor. Then the downstream signaling transduction induced by HER2-containing heterodimers could be reduced [99,100,101]. The first generation HSP90 inhibitor tanespimycin (17-AAG, KOS-953; Bristol-Myers Squibb, USA), has shown active antitumor activity against HER2+ BC. The therapeutic efficacies of both phase I study of tanespimycin plus trastuzumab and a subsequent single-arm phase II trial were encouraging [102,103]. Furthermore, the other two HSP90 inhibitors, retaspimycin (IPI-504; Infinity Pharmaceuticals) and AUY922 (Novartis), are currently under early phase clinical evaluation as single agents or in combination with trastuzumab [13].

Inhibitors of downstream signaling pathways are also a class of emerging agents against HER2+ BC. These inhibitors, which include mammalian target of rapamycin (mTOR) inhibitors and PI3K inhibitors, act directly on downstream signaling pathways to inhibit tumor cell proliferation and promote apoptosis [13,14,68]. Preclinical data demonstrated that the use of these inhibitors may provide a solution for the acquired resistance of anti-HER2 therapy by inhibiting the upregulation of downstream signaling transductions. Everolimus is an example of these inhibitors which is thought to be the first non-HER2 targeted agent to specifically address the proposed underlying resistance mechanism caused by trastuzumab [62].

### 4.5. Combination Therapy

Strategies of combining HER2 targeted agents with each other and combining anti-HER2 drugs with chemotherapy are usually carried out for the treatment of HER2+ BC. Various therapeutic approaches have been developed [104]. Combination therapies using anti-HER2 monoclonal antibodies and chemotherapeutic agents are the major approaches for treating HER2 positive breast cancer, especially for metastatic cancer [105]. Lapatinib was also used in combination with chemotherapy drugs such as paclitaxel, anthracyclin and docetaxel [106]. Furthermore, trastuzumab in combination with lapatinib can also dramatically improve the ORR and clinical benefit rate for the treatment of HER2+ BC patients [107,108]. This combination therapy is also commonly used in clinic for HER2+ BC therapy. In order to achieve higher efficacy with lower side effects, more therapeutic strategies should be explored and developed.

## 5. Anti-HER2 Molecular Therapy for Breast Cancer with HER2 Somatic Mutations

Recent studies reported that activating mutations of HER2 in patients without HER2 amplification might occur in 2%–4% of breast cancers [109]. These HER2 somatic mutations are caused by formation of intracellular HER2 residues which makes the tyrosine kinase constitutively active. They are likely to drive HER2-dependent tumor progression, though patients with HER2 mutant breast cancer could not be detected by IHC or FISH test [110,111,112]. Some studies showed that patients with activating HER2 mutations might also benefit from anti-HER2 targeted agents, especially for neratinib. For example, Bose and co-workers found that seven activating mutations in their experimental system were sensitive to neratinib [113]. A more recent study also pointed out that neratinib could inhibit the growth of cell lines with mutated HER2, and early clinical data of neratinib acting on HER2 mutant breast cancer were promising [114]. However, not all cases support the point of view that HER2 targeted therapy is beneficial. Some studies also indicated that patients with HER2 mutations could show resistance to lapatinib [109,113,115]. Thus, it remains uncertain whether HER2 somatic mutations can be targets for anti-HER2 molecular therapy. There is continuous ongoing research about this.

## 6. Conclusions

HER2 is one of the best characterized molecular biomarkers and an ideal target for developing therapeutic strategy for the treatment of HER2+ BC. Clinically, methods for evaluating HER2 status are generally utilized to determine the subtype of breast cancers, and then identify the appropriate molecular therapy for corresponding breast cancer. Based on the understanding of the HER2 biology and its role in breast cancer, a variety of HER2 targeted agents have been developed. Among those agents, monoclonal antibody trastuzumab as the prior choice for the treatment of HER2+ BC benefits patients significantly in clinical practice. However, intrinsic or acquired drug resistance to trastuzumab may be engendered in many patients with HER2+ BC. Therefore, clinically, combination therapies of trastuzumab and other anti-HER2 agents and combination therapies of anti-HER2 agents and chemotherapy are commonly conducted to optimize their therapeutic efficacy. However, these combination therapies still have some limitations, such as side effects. The economic costs of combination of HER2 targeted therapies may be prohibitive for patients. This means that only a minority of patients can gain benefits from the combination. The ultimate purpose is to be able to customize or personalize drug therapy with high efficacy and low toxicity. Hence, a great deal of effort is still needed to seek better therapeutic strategies to combat HER2+ BC.

## Figures and Tables

**Table 1:** Scoring criteria for immunohistochemistry (IHC) results [51].

IHC Score	Represent	Numbers of HER2 Receptors	Staining Pattern	Percentage of Cells Stained
0	Negative	<20,000	No staining	0
1+	Negative	about 100,000	Faint incomplete staining	<10%
2+	Weak positive	about 500,000	Light to moderate complete staining	>10%
3+	Strong positive	about 2,300,000	Strong complete staining	>30%

**Table 2:** Recommended reporting of FISH results [51,53].

Amplification for HER2	Dual-Probe		Single Probe
	HER 2/CEP 17	HER2 Signals/Cell	HER2 Signals/Cell
Positive	≥2.0	≥4.0	≥6.0
	≥2.0	<4.0	
	<2.0	≥6.0	
Equivocal	<2.0	≥4.0 and <6.0	≥4.0 and <6.0
Negative	<2.0	<4.0	<4.0
